# Clostridium Collagenase Impact on Zone of Stasis Stabilization and Transition to Healthy Tissue in Burns

**DOI:** 10.3390/ijms22168643

**Published:** 2021-08-11

**Authors:** Rosanne E. Frederick, Robert Bearden, Aleksa Jovanovic, Nasreen Jacobson, Rajiv Sood, Sandeep Dhall

**Affiliations:** Smith & Nephew Plc., Fort Worth, TX 76107, USA; Aleksa.Jovanovic@smith-nephew.com (A.J.); Nasreen.Jacobson@smith-nephew.com (N.J.); rajiv.sood@burncenters.com (R.S.); sandeep.dhall1@gmail.com (S.D.)

**Keywords:** collagenase, enzyme debridement, burn conversion, zone of stasis

## Abstract

Clostridium collagenase has provided superior clinical results in achieving digestion of immediate and accumulating devitalized collagen tissue. Recent studies suggest that debridement via Clostridium collagenase modulates a cellular response to foster an anti-inflammatory microenvironment milieu, allowing for a more coordinated healing response. In an effort to better understand its role in burn wounds, we evaluated Clostridium collagenase’s ability to effectively minimize burn progression using the classic burn comb model in pigs. Following burn injury, wounds were treated with Clostridium collagenase or control vehicle daily and biopsied at various time points. Biopsies were evaluated for factors associated with progressing necrosis as well as inflammatory response associated with treatment. Data presented herein showed that Clostridium collagenase treatment prevented destruction of dermal collagen. Additionally, treatment with collagenase reduced necrosis (HMGB1) and apoptosis (CC3a) early in burn injuries, allowing for increased infiltration of cells and protecting tissue from conversion. Furthermore, early epidermal separation and epidermal loss with a clearly defined basement membrane was observed in the treated wounds. We also show that collagenase treatment provided an early and improved inflammatory response followed by faster resolution in neutrophils. In assessing the inflammatory response, collagenase-treated wounds exhibited significantly greater neutrophil influx at day 1, with macrophage recruitment throughout days 2 and 4. In further evaluation, macrophage polarization to MHC II and vascular network maintenance were significantly increased in collagenase-treated wounds, indicative of a pro-resolving macrophage environment. Taken together, these data validate the impact of clostridial collagenases in the pathophysiology of burn wounds and that they complement patient outcomes in the clinical scenario.

## 1. Introduction

The theory of burn wound conversion refers to the phenomenon of progressive tissue necrosis from the zone of coagulation to the zone of stasis after cessation of the initial injury [1]. Progressing tissue damage represents an increase in total burn surface area and depth, leading to greater risk of local and systemic complications, and patient mortality [2]. Emanating from the devitalized zone of coagulation, secondary necrosis of partial-thickness to deep or full-thickness wounds is largely attributed to decreased vascular perfusion and an associated inflammatory response within the zone of stasis. Appropriate treatment of these burns represents a unique challenge to both experienced and inexperienced medical professionals due to the complexity of wound assessment and ongoing pathophysiology, risking delays in therapy to prevent additional tissue loss. Current clinical burn care is directed at treatment modalities to prevent this conversion of the burn wound. These modalities include appropriate resuscitation, management (either surgical or non-surgical) of the burn wound, attention to nutrition, repletion and management of hyper-metabolic response. To further treatment strategies, clinical and scientific research in recent years has focused on elucidating the pathophysiology of burn wound conversion theory with the aim of developing associated therapeutic interventions. Although minor achievements have been made [3,4], the translation to clinical practice has not yet been realized.

The progression of tissue death from burns is a result of chemical and mechanical mediators altering local cell viability. Cell death is classically characterized by a biochemical mechanism of either apoptosis or oncosis. Apoptosis occurs as a result of intrinsic cell stress or extrinsic signals in the microenvironment and persists in burns, correlating with local vascularity and perfusion status [5]. Considered an organized, programmed cell death, apoptotic cells condense organelles in preparation for phagocytosis, thereby minimizing the inflammatory response in the environment surrounding the dying cell. Conversely, oncosis, also termed necrosis, is an accidental, passive release of internal cellular contents and is observed to be the prominent mechanism of cell death in burn injuries [6,7]. The swelling and subsequent loss of cell wall integrity leads to a dispersion of intracellular organelles to the environment outside the cell, resulting in induction of a substantial influx of inflammatory mediators to the surrounding tissue. While inflammation mitigates infection risk and is necessary for functional wound healing, the continued presence of debris, pro-inflammatory cytokines and free radicals is proven to be detrimental and leads to burn injury progression with a pattern of fibrosis and increased scarring [8,9,10,11,12,13,14,15,16]. Moreover, tissue necrosis is exacerbated by increased vessel occlusion [17], limited monocyte diapedeses [18], delayed neutrophil efferocytosis [19] and the prolonged presence of a classic activated macrophage, or M1, response [18]. Therefore, it is imperative that devitalized tissue and inflammation are effectively managed following burn injuries to minimize secondary necrosis.

Currently, debridement and grafting are considered the standard of care to mitigating inflammation and ischemia, and reduce the risk of infection and scarring [20,21,22]. Removing necrotic tissue has multiple benefits in the resolution of a burn injury, such as eliminating the nutrient source for microbial growth, extenuating the extended immune response, minimizing the damage to microvascular network by cellular constituents, preventing delays in clinical assessment and allowing for a more rapid re-epithelialization. The abovementioned benefits support the improvement of wound bed health and aid in preparation for secondary therapies. Historically, there are two approaches to debridement: (1) early tangential excision to sequentially remove layers of devitalized tissue; and (2) application of topical agents to support local debridement and allow for wound demarcation. One such topical agent commonly utilized in clinical practice is enzymatic debridement via clostridial collagenase, derived from *Clostridium histolyticum*. Clostridial collagenase has been shown to aid in the continued removal of immediate and accumulating necrotic tissue while leaving healthy tissue unharmed [23,24]. While clinical utility of clostridial collagenases has been well documented as showing superior results as compared to other topical agents [25,26,27], recent studies have revealed the unique ability of clostridial collagenase and its degradative by-products to modulate cellular response, thereby fostering an anti-inflammatory milieu [28]. Specifically, debriding with clostridial collagenases influences macrophage polarization from a pro-inflammatory M1 state to a reparative, or M2, state in acute and chronic diabetic wounds [29]. The pro- to anti-inflammatory switch allowed for the stimulatory secretion of pro-resolution-associated cytokines and growth factors. In addition, collagen cleavage byproducts produced via clostridial collagenase debridement [30] contribute to the chemotaxis and proliferation of fibroblasts [31], keratinocytes [32] and endothelial cells [31].

The objective of this study is to shed light on the benefits of clostridial collagenase debridement in the treatment of burn wounds and its impact on limiting burn injury conversion. We hypothesized that collagenase treatment provides an environment to promote tissue in the zone of stasis in a burn wound to stabilize a transition to healthy tissue rather than expansion of apoptosis and necrosis, which can ultimately cause damage to deeper tissue and propagate further destruction from the original burn. Understanding of the cellular and molecular mechanism will provide the scientific rationale to select and use clinically relevant options to halt wound progression and preserve tissue that may have otherwise been lost due to secondary necrosis or aggressive surgical debridement.

## 2. Results

**Figure 1:**
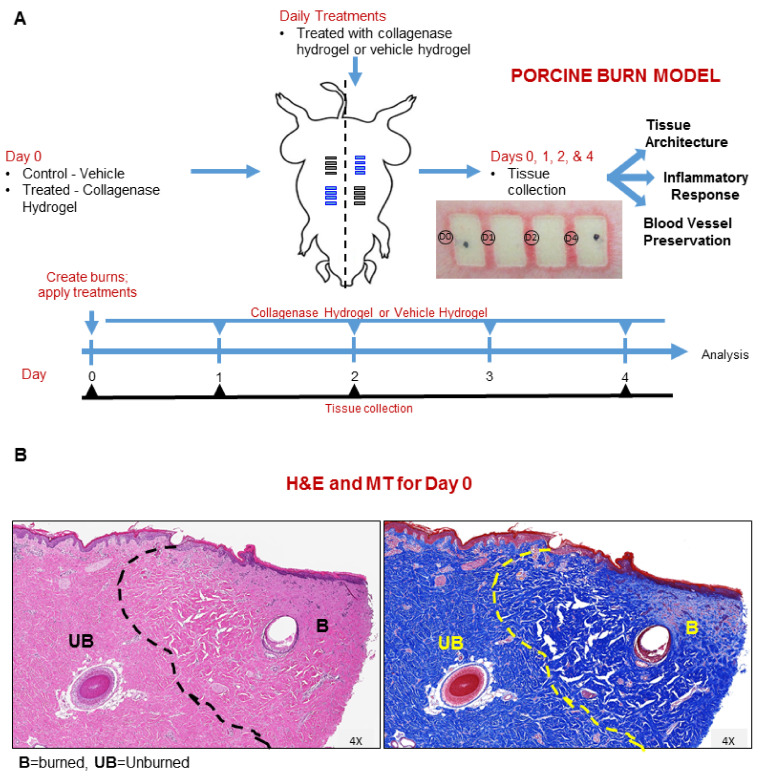
Burn study design schematic and treatment protocol. (**A**) Animals received Clostridium collagenase treatment daily. Tissues were collected at 0, 1, 2, 4 and 7 days post-burn from different burn wound interspaces from comb burns on two pigs and processed for histology. Black bars indicate location for vehicle hydrogel treatment and blue bars indicate collagenase hydrogel treatment location on porcine drawing. (**B**) Representative H&E and MT section stains for day 0 tissues collected. Areas of burned ‘B’ and unburned ‘UB’ tissue are indicated to demonstrate the focus area at the burn edge.

To investigate the impact clostridial collagenase has on the zone of stasis in burn wounds, we used a brass comb burn porcine model to create a total of 16 burn injuries in two animals (2 animals × 8 burns × 2 treatments) (Figure 1A). Half of the comb burns for each pig were treated daily with Clostridium collagenase hydrogel or vehicle hydrogel (control) and the wound interspaces were biopsied (four biopsies, two from each pig, for each treatment at designated time points) for various histopathology and immunohistochemistry analyses used to assess the burn wound progression. Figure 1B shows the hematoxylin–eosin (H&E) staining, revealing an expansion of the burn-damaged area away from the burn edge and a well-defined junction between burned and unburned tissue. The Masson’s trichrome (MT) blue staining similarly revealed expanded damage in dermal collagen architecture, where the blue collagen stain had disappeared and the general collagen network had collapsed (Figure 1B).

### 2.1. Clostridium Collagenase Facilitates Early Epidermal Separation and Loss

**Figure 2:**
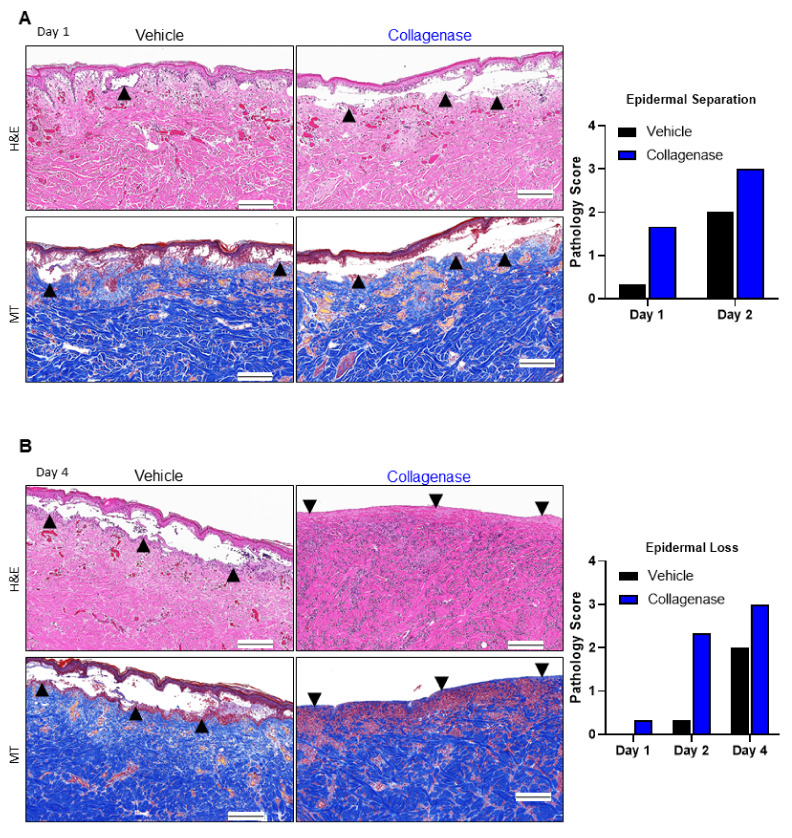
Collagenase-treated burns show early epidermal separation and epidermal loss. Histological analyses of burn comb interstices in pigs via hematoxylin–eosin (H&E) and Masson’s trichrome (MT) stains. (**A**) H&E and MT stains showed early epidermal separation relative to vehicle-treated wounds, indicated by black arrows. (**B**) H&E and MT stains showed early epidermal loss relative to vehicle-treated wounds, indicated by black arrows. For the histopathology grading system, see Table 1. Black scale bars represent 200 μm. Pathology scores for 4 biopsies per time point and per treatment (*n* = 4) were averaged and the columns represent the average scores.

Further histological examination of H&E-stained sections of the harvested skin revealed a greater magnitude of epidermal separation for collagenase-treated pigs relative to control on days 1 and 2 (Figure 2A). Furthermore, damaged epidermal slough was observed earlier in the study and to completion in collagenase-treated pigs relative to control (Figure 2B). MT-stained sections of collagenase-treated burns were more consistent with an intact interwoven collagen network, indicative of improved preservation of extracellular matrix structure. In contrast, control burns showed diminished collagen blue staining and continued disintegration of the fibrillary collagen structure over time.

### 2.2. Clostridium Collagenase Reduces Necrosis

**Figure 3:**
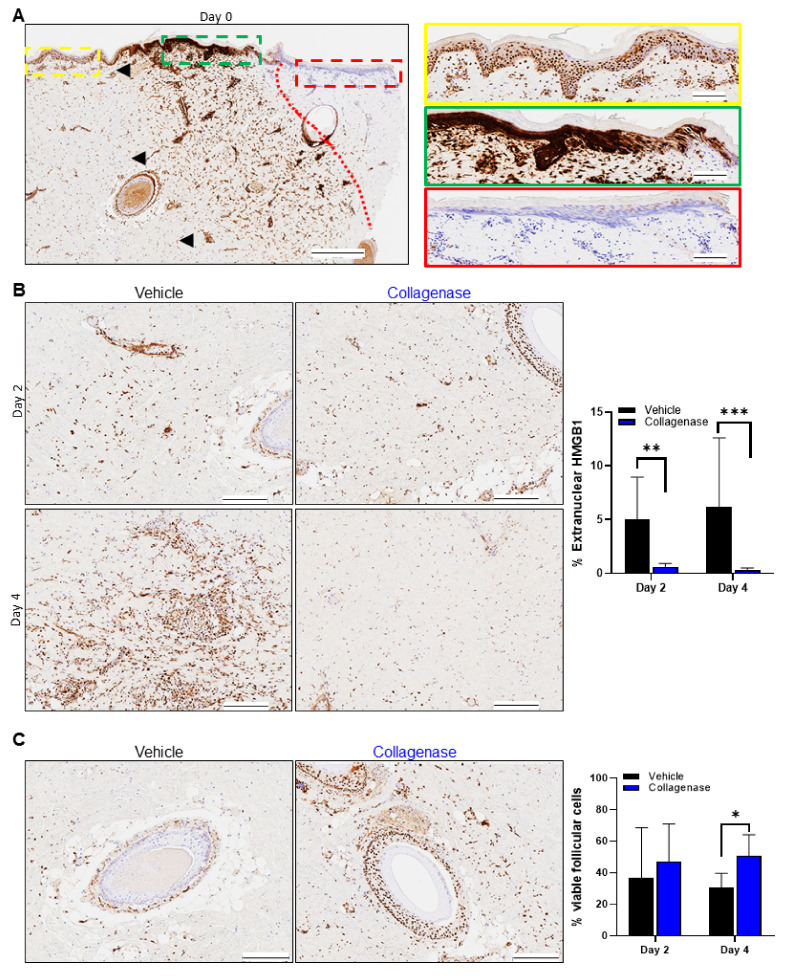
Collagenase-treated burns limit necrosis. HMGB1 staining pattern in porcine burn interspaces for various days in wounds treated with vehicle and collagenase hydrogels. (**A**) Tissue biopsy from day 0 shows the border between positive and negative staining for HGMB1, indicated by the red dotted line. Left panel: black arrows indicate the wound edges with normal skin, including normal dermis and epidermis present in burn interspace of the tissue section, and black bar represents 500 µm. Right panel: a horizontal strip was selected along the epidermal–dermal junction of the wound and divided into three magnified images from left to right (from yellow, to green, and to red dashed border) to visualize the specific staining patterns observed. (**A**, yellow) Image showing all viable tissue with strongly positive nuclear HMGB1 staining. (**A**, green) Diffuse cytosolic and extracellular HMGB1 staining released from necrotic cells. (**A**, red) The dermis directly under burn wound is observed to not have any positive HMGB1 staining and all nuclei are only stained blue. Black scale bars in yellow, green and red stained images represent 100 μm. (**B**) HMGB1 staining pattern of wound interspace sections on days 2 and 4 for vehicle and collagenase treatments. Collagenase treatment showed HMGB1 staining pattern with significantly more nuclear HMGB1 stain, indicative of preserved cell viability, relative to treatment with vehicle that showed diffuse extracellular and cytoplasmic HMGB1 staining, indicating expansion of the burn zone in vehicle-treated burns. Black scale bars represent 200 μm. Percent (%) extranuclear HMGB1 was calculated from the total number of positive nuclear HMGB1/total cells as shown by hematoxylin blue stain × 100%. Values represent mean of 4 biopsies per time point and per treatment (*n* = 4) ± SD. ** *p* < 0.01; *** *p* < 0.001. (**C**) HMGB1 staining pattern of hair follicles in the wound interspace sections on day 4 for vehicle and collagenase treatments. A diffuse extracellular and cytoplasmic HMGB1 staining along with complete absence of stain is shown for vehicle-treated wounds, while treatment with collagenase shows significantly greater strongly positive nuclear HMGB1 staining. Black scale bars represent 200 μm. Percent (%) viable follicular cells was calculated from the total number of positive nuclear HMGB1 follicular cells/total follicular cells as shown by hematoxylin blue stain × 100%. Values represent mean of 4 biopsies per time point and per treatment (*n* = 4) ±SD. * *p* < 0.05.

To confirm the impact on necrosis in vivo, we stained the burn wound sections for High Mobility Group Box protein 1 (HMGB1). Figure 3A shows HMGB1 staining on day 0, revealing the necrotic region immediately post-burn and the expansion of the burn-damaged area away from the burn edge, as observed for the H&E and MT staining, and a well-defined junction between burned and unburned tissue, representing the transition of burn injury from the zone of coagulation to the zone of stasis. The HMGB1 staining pattern consisted of the absence of stain, indicating necrosis at the burn edge (Figure 3A, red box), which transitioned to diffuse extracellular staining from the burn edge and concentrated extracellular and cytoplasmic staining (Figure 3A, green box) immediately after burn injury. Beyond this area, HMGB1 showed strong nuclear staining, indicative of cell viability furthest away from the burn edge (Figure 3A, yellow box).

Via quantitative image analysis, measurement of the nuclear HMGB1 staining for collagenase-treated burns showed a continuous maintenance and improvement in the tissue of the zone of stasis on days 2 and 4, with an HMGB1 staining pattern consistent with preservation of the tissue surrounding the burn edge (Figure 3B, right panel). However, significantly more extranuclear HMGB1 stain was present in vehicle-treated burns on days 2 and 4, coupled with architectural distortion and nuclear streaming, indicating progression of necrosis and expansion of the burn zone (Figure 3B, right panel, graph). At the clinical level, hair follicle intactness is often a tool for assessing prognosis and ultimate burn care strategy, as intact hair follicles include essential multipotent stem cells that play a critical role in epidermal homeostasis, turnover and maintenance and are supportive in burn wound healing [2,22,33,34]. The comb burns treated with vehicle also demonstrated evidence of hair follicle necrosis (Figure 3C), and while there was evidence of hair follicle necrosis in both collagenase and vehicle-treated burns, it was less apparent in collagenase-treated burns relative to vehicle. By day 4, hair follicle preservation was significantly greater in collagenase-treated burns as compared to vehicle-treated burns (Figure 3C, graph).

### 2.3. Clostridium Collagenase Preserves and Facilitates Blood Vessel Recovery and Formation

**Figure 4:**
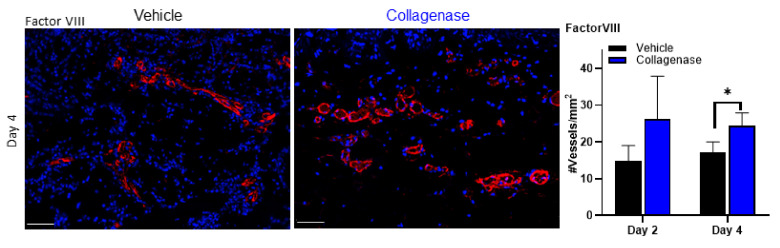
Collagenase-treated burns show blood vessel preservation. Factor VIII expression and blood vessel quantification in porcine burn interspaces in wounds treated with vehicle and collagenase hydrogels. Factor VIII expression in the wound interspace sections on day 4 show treatment with collagenase has significantly greater factor VIII expression, which translated to significantly greater blood vessel quantification per tissue area for collagenase-treated wounds. White scale bars represent 50 μm. Number of vessels/mm^2^ was calculated from the total number of vessels/total area in mm^2^ as visualized by anti-factor VIII stain. Values represent mean of 4 biopsies per time point and per treatment (*n* = 4) ± SD. * *p* < 0.05.

Blood vessel analysis was performed using the endothelial marker factor VIII. Damage to microvasculature of burn wounds is thought to mediate secondary tissue damage observed in burn wound progression [4,35]. While an overall increase in blood vessel density preservation was observed in collagenase-treated tissues by day 2, a significant increase in counts was observed over vehicle-treated tissues by day 4 (Figure 4).

### 2.4. Treatment with Clostridium Collagenase Limits Apoptosis

**Figure 5:**
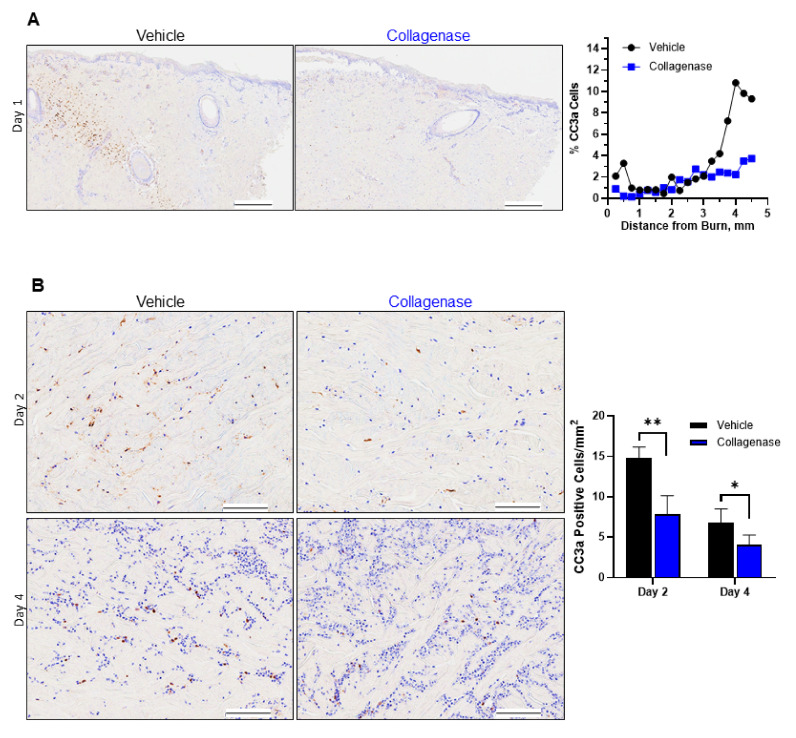
Collagenase-treated burns show limited apoptosis. Cleaved caspase 3a (CC3a) staining pattern in porcine burn interspaces on various days in wounds treated with vehicle and collagenase hydrogels. (**A**) Tissue biopsies from day 1 showed collagenase prevented burn-induced apoptosis in the burn wound surrounding area, while there was a significant increase in apoptosis observed in the wound interspaces for vehicle-treated burns. Percent (%) CC3a cells was calculated from the total number of positive stained CC3a cells/total cells as shown by hematoxylin blue stain × 100%. Black scale bars represent 500 μm. (**B**) CC3a staining pattern of wound interspace sections on days 2 and 4 for vehicle and collagenase treatments. Collagenase treatment showed CC3a staining pattern with significantly less CC3a stain, indicative of limitation of burn-induced apoptosis, relative to treatment with vehicle that showed greater CC3a staining, indicating increase in apoptosis in the burn interspaces of vehicle-treated wounds. Black scale bars represent 100 μm. Number of CC3a-positive cells/mm^2^ was calculated from the total number of CC3a-positive cells/total area in mm^2^ as visualized by CC3a stain. Values represent mean of 4 biopsies per time point and per treatment (*n* = 4) ± SD. * *p* < 0.05; ** *p* < 0.01.

Apoptosis was assessed with antibody stain for cleaved caspase 3a (CC3a), a well-known marker for apoptotic committed cells [36]. As shown in Figure 5A, early cell apoptosis was not observed at the burn edge for either collagenase- or control-treated burns. However, on day 1, a band of cells with positive CC3a staining was observed deep to the area of necrosis for vehicle-treated tissues, indicating a large number of apoptotic cells at the interface of necrotic and viable tissue. While collagenase-treated tissue experienced apoptosis in a similar region, the observed quantity of apoptotic cells was significantly reduced (Figure 5A, graph). Similarly, on days 2 and 4 of the study, collagenase-treated tissues had significantly fewer apoptotic cells present relative to vehicle-treated tissues (Figure 5B, graph).

### 2.5. Clostridium Collagenase Leads to Early Neutrophil Recruitment

**Figure 6:**
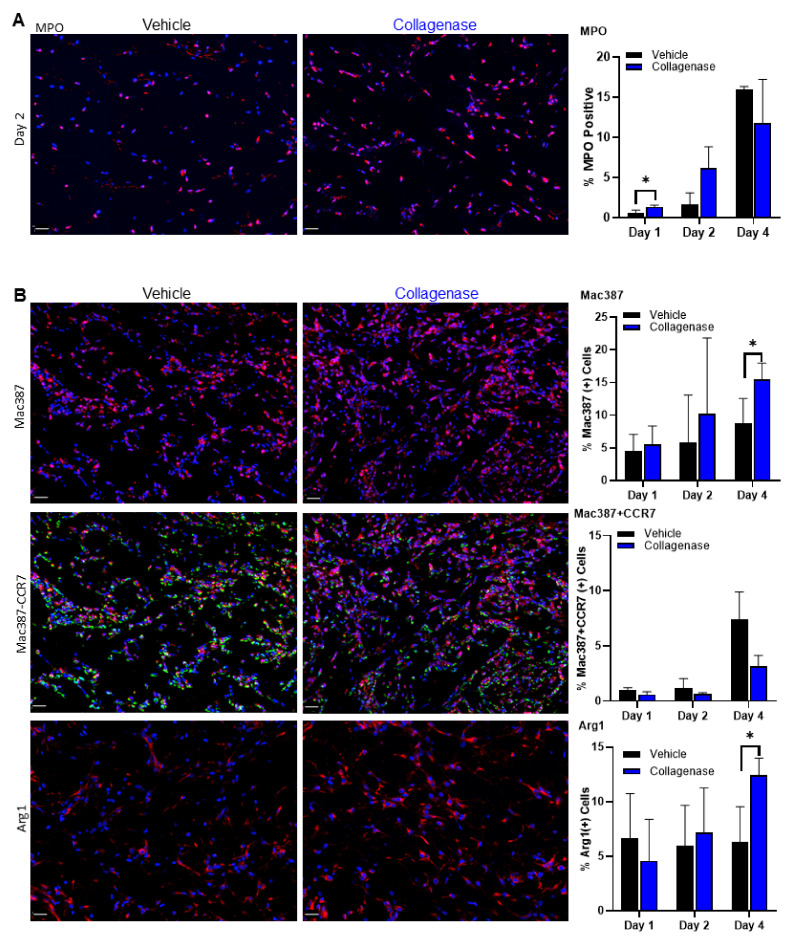
Collagenase-treated burns show early and improved inflammatory response. (**A**) Burn wound interspace sections were immunolabeled with antibodies against myeloperoxidase (MPO), an Alexa Fluor 555-labeled neutrophil marker. Early induction of neutrophils in collagenase-treated burns was observed 1 and 2 days post-burn. The number of neutrophils was counted for each tissue cross-section and %MPO-positive cells was calculated from the total number of positive stained MPO cells/total cells × 100%. (**B**) Immunolabeled monocytes and macrophages in the wound interspace sections on day 4 for vehicle and collagenase treatments. Top panel: the number of macrophages was labeled for each tissue cross-section with Alexa Fluor 555-labeled macrophage marker Mac387 and %Mac387-positive cells was calculated from the total number of positive stained Mac387 cells/total cells × 100%. Middle panel: the M1 macrophage phenotype was immunolabeled with M1 macrophage marker CCR7 and was depicted with the dual stain for macrophage marker Mac387 with the level of M1 macrophage phenotype calculated for each tissue cross-section with markers for regions visible in the Alexa 555 range identified as Mac387-positive staining, and regions visible in the Alexa 647 range identified as CCR7-positive staining. A cell was considered to be dual positive if both Mac387- and CCR7-positive stainings were present in the cytoplasm and the M1 macrophage phenotype was calculated for each tissue cross-section and %Mac382 + CCR7 positive cells was calculated from the total number of dual positive stained Mac382 + CCR7 cells/total cells × 100%. Bottom panel: the number of M2 macrophages was labeled for each tissue cross-section with Alexa Fluor 555-labeled macrophage marker Arg1, the level of M2 macrophage phenotype was calculated for each tissue cross-section with a marker for arginase 1 (Arg1), and %Arg1 positive cells was calculated from the total number of positive stained Arg1 cells/total cells × 100%. For all images, total cells were counted by DAPI blue nuclear stain. White scale bars represent 20 μm. Values in all graphs represent mean of 4 biopsies per time point and per treatment (*n* = 4) ±SD. * *p* < 0.05.

Myeloperoxidase (MPO) marker was detected earlier in collagenase-treated burns, indicating a robust neutrophil response, with an attenuated increase on days 2 and 4 (Figure 6A). In contrast, vehicle-treated tissues had a delayed neutrophil response relative to collagenase-treated burns and intensified influx of neutrophils was observed on day 4, consistent with continued inflammation and damage due to the process of necrosis, observed with the HMGB1 staining, and leading to a delayed healing phenotype.

### 2.6. Infiltration of Inflammatory Cells Is Diminished with Clostridium Collagenase Treatment

Further investigation of the inflammatory response showed the recruitment of monocytes and macrophages to the site of injury. While this cellular recruitment was observed for both collagenase- and vehicle-treated burns, collagenase-treated wounds tended toward a greater monocyte and macrophage recruitment throughout days 1, 2 and 4, as visualized by Mac387 staining (Figure 6B, top panel). A closer look using the CCR7 marker for M1 macrophage phenotype (Figure 6B, middle panel) and the arginase 1 marker for M2 macrophage phenotype (Figure 6B, bottom panel) showed a concomitant decrease in M1 signaling and a significant increase in M2 signaling for collagenase-treated burns by day 4 than that observed for vehicle-treated burns. The diminishing M1 phenotype parallel to M2 phenotype influx for collagenase-treated burns indicates a transition out of the pro-inflammatory response observed on earlier days in this study.

## 3. Discussion

Prevention of the consequences of burn progression, as it relates to the salvage of tissue in the zone of stasis, is of vital importance in clinical practice. Changes in permeability of the epithelium and subsequent inflammatory responses epitomize the earliest complications observed for severe thermal injury of the skin. Using the well-known burn comb model [37] allowed for an instrumental visual of the temporal resolution and spatial analysis of the hallmarks of burn injury, including evaluating (1) initial necrosis and subsequent apoptosis; (2) inflammatory response of initial neutrophil infiltration and succession of dynamic changes in macrophage infiltration; (3) effect of blood vessel preservation and recovery. Implementation of image analysis software was critical for objective analysis of the aforementioned signatures for burn injury, aiding in quantification of necrosis, apoptosis, blood vessel quantification and inflammatory cellular modulation. Image analysis for histological quantification has been revolutionized to avoid the effects of human subjectivity in visual evaluation [38,39,40]. We sought to better understand the impact clostridial collagenase debridement has on burn wounds and hypothesized that collagenase treatment prepares the burn environment to stabilize the zone of stasis, a critical area for burn progression of tissue surrounding the coagulated surface of a burn, and transition to healthy tissue rather than expansion of necrosis and apoptosis. Our study herein shows that the application of clostridial collagenase to porcine skin burn wounds greatly prevents the conversion of tissue in the zone of stasis via limiting necrosis and apoptosis. We further hypothesized that the mechanism of dampening conversion to necrotic tissue could be due to the impact of collagenase on tissue perfusion and markers of inflammation in response to burn injury. We show that an early epidermal separation and epidermal loss facilitated by collagenase treatment prevented destruction of dermal collagen, led to preservation of invaluable blood vessels and promoted an immunomodulatory response reflective of accelerated inflammatory resolution (Figure 2, Figure 3, Figure 4, Figure 5 and Figure 6). The impact observed in the burn microenvironment bolsters the observed positive clinical outcomes for burns treated with collagenase.

Early epidermal separation and subsequent epidermal loss of the burn interstices was observed for collagenase-treated samples, which had an apparent impact on collagen architecture preservation, as revealed by the H&E and MT staining (Figure 2A,B). There was also an observable influx of cells with a greater presence of cellular staining for collagenase-treated burns, which prompted further investigation of the impact of vessel occlusion and the inflammatory response of the burn interstices. Along with these morphological observations, there was also evidence of significantly less necrosis of the burn interspaces with decreased extranuclear detection of the necrotic marker HMGB1 in collagenase-treated tissues, as well as hair follicle preservation (Figure 3B,C). HMGB1 is a nuclear protein generally released from necrotic cells and its diffuse staining pattern is associated with burn wound progression and zones of necrosis [7,41,42]. Day 4 analyses of the nuclear HMGB1 staining pattern demonstrated that collagenase-treated burns continued the observed maintenance of the tissue architecture, while diffuse extracellular and cytoplasmic HMGB1 staining showed an expansion and associated necrosis of the identified burn zone of stasis for vehicle-treated burns. The viable cells maintained in hair follicles are an indicator that progressing necrosis was muted by collagenase treatment, which was not observed for vehicle-treated tissues, as there was significant evidence of continued necrosis in these tissues up to day 4.

We also investigated the impact collagenase treatment had on another mechanism of cell death in the burn wound microenvironment—apoptosis. Apoptotic cell death was differentiated from necrotic cell death as being characterized by cytoplasmic staining with a cleaved caspase 3a (CC3a) marker and was detected in cells at the boundary between necrotic and viable tissue, as has been observed in previous studies on burn progression [6,36,43], with collagenase-treated tissues demonstrating a limitation of apoptosis coinciding with the limitation of necrosis (Figure 5A). Days 2 and 4 continued to demonstrate the significant evidence of continued tissue preservation for collagenase-treated burns with limited apoptotic cell commitment (Figure 5B). The limitation of apoptosis indicated that collagenase treatment had a considerable impact on conserving the zone of stasis.

Another component of the assessment of burn prognosis that is instrumental to salvaging the zone of stasis is the preservation of blood vessels, with severity of tissue destruction dependent on loss of blood flow [22]. It has also been reported that in the zone of stasis, hypoxia and ischemia, as a result of diminished blood flow, can lead to tissue necrosis [44]. We sought to understand the impact collagenase treatment had on blood vessel integrity using factor VIII [45,46,47,48]. We found that collagenase-treated tissues had significantly greater blood vessel preservation throughout days 2 and 4 (Figure 4). Again, computed image analysis was instrumental in the assessment of blood vessel quantification and investigating the remaining vasculature in these tissues. The presence of conserved blood vessels in burn wounds is imperative in the exchange of nutrients, immune cells and oxygen in healing wounds [49]. Our findings suggest collagenase application has the advantage in these early stages following burn injury in preventing continued damage due to the process of necrosis that often leads to a significant delay in healing.

Preservation of the tissue interspaces with respect to limited necrosis and apoptosis in collagenase-treated burns prompted us to further investigate the impact of collagenase treatment on the inflammatory response in the zone of stasis. We found that collagenase treatment allowed for an earlier neutrophil infiltration relative to vehicle treatment as observed significantly on day 1 with an attenuated increase on days 2 and 4 (Figure 6A). Early influx of neutrophils has been shown to lead to faster epidermal disjunction and enhanced wound healing [50]. Additionally, neutrophils play a prominent role in the process of tissue regeneration after injury, related to the phagocytosis of necrotic material and the recruitment of other inflammatory cells [51]. Indeed, earlier recruitment of macrophages was observed for collagenase-treated burns, significantly evident over vehicle-treated burns by day 4 (Figure 6B, top panel). To understand the dynamic and heterogenic response of the macrophage influx, we targeted both the CCR7 marker for M1 macrophages associated with a pro-inflammatory phenotype and the arginase 1 marker for M2 macrophages associated with an anti-inflammatory phenotype [52,53,54,55]. The M1 macrophage presence was similar on days 1 and 2 for both vehicle- and collagenase-treated burns. However, the pro-inflammatory macrophage phenotype in collagenase-treated wounds trended downward, indicating a faster clearance relative to vehicle-treated wounds (Figure 6B, middle panel). When probed for the anti-inflammatory M2 macrophage phenotype, there was a significant increase by day 4 in collagenase-treated burns, underscoring the evidence that collagenase treatment reduces inflammation, allowing for the influx of monocytes known to release anti-inflammatory cytokines (Figure 6B, bottom panel). It is imperative that the inflammation following burn injuries is effectively optimized to balance the benefits and concurrently prevent an extended response with burn progression and subsequent development of scarring and fibrosis [56]. The shift to an M2 phenotype with collagenase treatment suggests a faster progression away from inflammation, known to stimulate angiogenesis, coinciding with the significantly greater blood vessel density observed by day 4 and indicates a progression through the healing cascade. This phenomenon was similarly observed in a previous study where collagenase elicited a substantial induction in arginase 1, M2 marker genes, IL-10 and CD206 in a murine wound model [29].

Clinically, the use of collagenase on the burn wound allows a much earlier determination of the potential need for surgical intervention. Using the clinical parameter of completed burn would healing within a 2–3-week window from the time of injury, decisions on the need for surgery are more definitive with the use of collagenase as primary treatment for partial-thickness to deep partial-thickness wounds (unpublished data). The absence of eschar/pseudoeschar and better visibility of the injured wound bed with the use of collagenase allows for the intervention of operating earlier or holding off surgical intervention to allow endogenous wound closure. This approach can have a long-term impact as previous studies have presented that treatment with clostridial collagenase reduced keloid and hypertrophic scarring associated with burn injuries [57]. As we collate these results, we have begun the process of elucidating the positive outcomes from previous clinical publications. Treatment with collagenase resulted in reduced time to complete debridement and trajectory for healing potential [25,58,59,60]. In tandem, collagenase treatment allowed for earlier cellular influx throughout the interstitial space and vascular perfusion. Further, these results lead to the finding of immunomodulation and preservation of tissue. These findings are important in the burn space as patients are affected for life, oftentimes needing additional clinical interventions to address scar management, contracture and cosmesis.

Our study is not devoid of limitations, which should be addressed in future studies. First, while the effects on the impact of collagenase treatment on the zone of stasis of burns was assessed, the molecular mechanisms by which collagenase affects the burn zones remain unclear. A subject of further studies could be investigated regarding the impact collagenase has on inflammatory markers and the pathways that regulate these factors during burn injury. Another limitation was the number of specimens used for each time point. Increasing the number of specimens would have added to the robustness of the study herein. In the future, studies should include more specimens per designated time point.

## 4. Materials and Methods

### 4.1. Materials and Reagents

#### 4.1.1. Test Articles

The active ingredients in Collagenase Santyl Ointment (CSO) are clostridial collagenases that are produced by Clostridium histolyticum bacteria via a proprietary fermentation process and have been widely used in biomedical research to dissociate tissues, isolate cells and as a therapeutic drug for the removal of necrotic wound tissues since 1965 [31,32,61,62]. The active constituent of CSO, henceforth called Clostridium collagenase, is a powder, as opposed to CSO that is formulated in a hydrophobic ointment base suited for human wound applications and not suitable for the fast healing and wound contraction observed in porcine wound applications. Therefore, a hydrogel formulation was optimized for use in this porcine burn model. Clostridial collagenase test articles were prepared using a proprietary method (Smith and Nephew, Fort Worth, TX, USA). A hydroxyethylcellulose (HEC)-buffered (pH = 7.40) hydrogel base was used as a delivery vehicle for clostridial collagenase (0.8% *w*/*w*). The control vehicle used was the same buffered hydrogel base.

#### 4.1.2. In Vivo Testing

Animals: Animal studies were approved by the BRIDGE PTS Institutional Animal Care and Use Committee (IACUC Code BPTS-18-02, and date of approval letter was June 6, 2018). Two (2) specific pathogen-free, female, commercially raised, Yorkshire-cross pigs (30–40 kg ± 5 kg) were obtained at the testing facility and allowed an acclimation period of at least 7 days prior to surgery. Animals were pair-housed, prior to the study, and cared for in accordance with the guideline in the Guide for the Care and Use of Laboratory Animals, published by the National Research Council and approved by AAALAC International. The pigs were fed antibiotic-free feed and tap water was provided ad libitum.

Burn Wound Model: Prior to burn injury, the pigs were pre-medicated by intramuscular injection of atropine (0.05 mg/kg) for 15 min followed by a brand of tiletamine–zolazepam (4.0–6.0 mg/kg) followed by mask inhalation of 0.5% to 5% isoflurane mixed with oxygen. Hair was removed from designated dorsa via clipper blades. The skin was prepared for surgery by wiping the surgical area with a chlorhexidine scrub and isopropyl alcohol in an alternating fashion 3 times to mimic skin preparation in humans. To relieve post-surgical wound pain, buprenorphine HCl (0.02 mg/kg; IM) was administered on day 0 and a fentanyl patch (50 µg/h) was secured to shaved skin on day 0 and replaced on day 3 for pain management.

Deep partial-thickness burn wounds were created using a custom-designed 620 g brass comb with four 20 mm × 10 mm prongs separated by 5 mm interspaces. Each brass comb was heated to 100 °C by immersion in water at a rolling boil and dried quickly prior to placing on the prepared skin surface for 30 s. The wounds were made parallel and to either side of the midline. Wounds were approximately 20 mm × 50 mm and were treated according to protocol (Figure 1). The 0.8% Clostridium collagenase hydrogel and vehicle hydrogel were applied edge-to-edge and spread evenly to the thickness of a nickel. The wounds were treated immediately post-burn (day 0) and then daily for seven days with secondary cover dressings that included a sterile non-adherent gauze (Curad^®^) and secured with a large Tegaderm^TM^ (3M) occlusive dressing. Wounds were wiped clean using saline-moistened gauze prior to sample acquisition and treatment applications.

#### 4.1.3. Immunohistochemistry

The immunohistological analyses were carried out for burn wounds, in which 8 mm punch biopsies were taken on a specified day post-burn from associated burn wound interspaces. Each 8 mm biopsy encompassed the 5 mm unburned interspace with 1.5 mm of burned tissue on either side (Figure 1A). For each time point, a total of 4 biopsies were extracted per treatment from two pigs. Collected wound tissue samples were fixed in 10% neutral buffered formalin. Tissue sectioning and staining were performed by Premier Laboratory (Boulder, CO, USA) using standard protocols for hematoxylin and eosin (H&E), and Masson’s trichrome (MT) staining. Cleaved caspase 3a (CC3a) and high mobility group box 1 (HMGB1) were stained using immunohistochemical (IHC) staining methods. IHC studies on skin wounds were conducted with anti-HMGB1 antibody for necrosis (Abcam, Waltham, MA, USA), anti-activated CC3a antibody for apoptosis (Cell Signaling, Boston, MA, USA). Von Willebrand factor/factor VIII, myeloperoxidase, Mac387, CCR7 and arginase 1 were stained using immunofluorescent (IF) staining methods. Blood vessels were stained by anti-factor VIII primary antibody (Dako, Burlington, ON, USA). Neutrophils were stained by anti-myeloperoxidase primary antibody (Abcam, Waltham, MA, USA). Monocytes and macrophages were stained with antibodies against Mac387 (Invitrogen, Waltham, MA, USA) with M1-macrophage marker CCR7 (Invitrogen, Waltham, MA, USA) for dual stain. The M2-macrophage phenotype was stained for anti-arginase 1 primary antibody (BD Biosciences, San Jose, CA, USA). Appropriate secondary antibodies were selected according to primary antibody species. Alexa Fluor 555 was used for Mac387, MPO and Arg1 positivity and Alexa Fluor 647 was used for CCR7 positivity.

The H&E- and MT-stained sections were graded through the examination of stained slides by a board-certified pathologist blinded to the treatments administered. A grading system to assess the epidermal separation and epidermal loss is shown in Table 1.

H&E-, MT-, CC3a- and HMGB1-stained slides were imaged at 20X using an Aperio ScanScope AT2 (Leica Biosystems, Lincolnshire, IL, USA) and IF-stained slides were imaged at 20X using a Vectra 3 Automated Quantitative Pathology Imaging System (Akoya Biosciences, Marlborough, MA, USA). Images for entire tissue sections under each condition were visualized under a microscope by experts in immunohistochemistry analysis who were not involved in this project and pre-agreed on intensity of the staining by looking at standardized slides, to provide image analyses. All image analyses were accomplished using programmable algorithms in HALO software (Indica Labs, Albuquerque, NM, USA)**.**

### 4.2. Statistical Analysis

GraphPad InStat Software (Graphpad, La Jolla, CA, USA) was used for statistical analysis and plotting graphs. Results are presented as mean ± SD. A Student’s *t*-test was used to determine the significance of differences between groups, whereby *p* < 0.05 was considered significant.

## 5. Conclusions

The data presented in this study truly begin to tell the greater story of the impact of clostridial collagenase in the pathophysiology of burn wounds and patient outcomes. Moreover, this study allows us to better understand the cellular mechanisms that complement the clinical scenario. Future studies should also examine the decreased risk of surgical intervention associated with deep partial-thickness burns as well as the acceptance of secondary grafting.

## Figures and Tables

**Table 1:** Histopathology grading system.

Epidermal Separation: Cleft between Dermis and Epidermis	
Grade	Description
0	Intact epidermis on entire section
1	Small area of epidermis separated from dermis (approximately less than 10% of section)
2	Mild, larger area of separation (approximately 10- 25% of section)
3	Moderate (approximately 25–75% of section)
**Epidermal loss: area of complete loss of epidermis**	
0	No epidermal loss
1	Small area (approximately less than 10%)
2	Larger area (approximately 10–25%)

## Data Availability

The data presented in this study are available in the article.
